# Basic leucine zipper transcription factor SlbZIP1 mediates salt and drought stress tolerance in tomato

**DOI:** 10.1186/s12870-018-1299-0

**Published:** 2018-05-08

**Authors:** Mingku Zhu, Xiaoqing Meng, Jing Cai, Ge Li, Tingting Dong, Zongyun Li

**Affiliations:** 1 School of Life Sciences, Jiangsu Normal University, 101 Shanghai Road, Xuzhou, Jiangsu Province 221116 People’s Republic of China; 2 Jiangsu Key laboratory of Phylogenomics & Comparative Genomics, Jiangsu Normal University, Xuzhou, Jiangsu Province People’s Republic of China

**Keywords:** Abscisic acid, Drought stress, Salt stress, *SlbZIP1*, Tomato

## Abstract

**Background:**

Basic region/leucine zipper (bZIP) transcription factors perform as crucial regulators in ABA-mediated stress response in plants. Nevertheless, the functions for most bZIP family members in tomato remain to be deciphered.

**Results:**

Here we examined the functional characterization of *SlbZIP1* under salt and drought stresses in tomato. Silencing of *SlbZIP1* in tomato resulted in reduced expression of multiple ABA biosynthesis- and signal transduction-related genes in transgenic plants. In stress assays, *SlbZIP1-*RNAi transgenic plants exhibited reduced tolerance to salt and drought stresses compared with WT plants, as are evaluated by multiple physiological parameters associated with stress responses, such as decreased ABA, chlorophyll contents and CAT activity, and increased MDA content. In addition, RNA-seq analysis of transgenic plants revealed that the transcription levels of multiple genes encoding defense proteins related to responses to abiotic stress (e.g. endochitinase, peroxidases, and lipid transfer proteins) and biotic stress (e.g. pathogenesis-related proteins) were downregulated in *SlbZIP1-*RNAi plants, suggesting that SlbZIP1 plays a role in regulating the genes related to biotic and abiotic stress response.

**Conclusions:**

Collectively, the data suggest that SlbZIP1 exerts an essential role in salt and drought stress tolerance through modulating an ABA-mediated pathway, and SlbZIP1 may hold potential applications in the engineering of salt- and drought-tolerant tomato cultivars.

**Electronic supplementary material:**

The online version of this article (10.1186/s12870-018-1299-0) contains supplementary material, which is available to authorized users.

## Background

Drought, salt and extreme temperatures are primary environmental factors that limit plant growth and reduce crop production [1]. Nevertheless, some plant species have developed diverse and complicated mechanisms at molecular, cellular and physiological levels that enable them to endure these unfavorable situations. Transcription factors (TFs) are pivotal regulatory proteins in signal transduction networks activated in plants response to various stresses [2]. Numerous studies have shown that plenty of such regulatory mechanisms generally follow the phytohormone ABA (abscisic acid)-dependent pathway. ABA has usually been deemed as the global plant stress hormone because of its remarkable effects on growth regulation during stress responses [3].

TFs often bind to specific cis-elements in the promoters of many genes to regulate their expression thereby enhancing stress tolerance [4]. Among numerous TF families, the bZIP TF family, known as ABA-response element binding factors (AREB) or ABF (ABRE-binding factors) has been isolated [5]. bZIP proteins are bipartite in structure containing DNA binding domains consisting of abundant basic amino acids that bind to DNA, and leucine zippers characterized by leucine residues spaced regularly at seven amino acid intervals [6]. Correlative research has indicated that ABA-dependent bZIP TFs generally bind to a conserved sequence with an ACGT core cis-element, especially A-box (TACGTA), ABRE (ACGTGG/TC), C-box (GACGTC) and G-box (CACGTG) [7, 8].

In plants, extensive documents have shown that bZIP genes participate in plenty of biological processes, including seed maturation, unfolded protein response, photomorphogenesis and light signaling, hormone signaling and organ development [9]. Furthermore, bZIP proteins also perform as crucial components in the signal transduction networks that mediate the response to various stresses, including salt, drought, cold and pathogen defense. Presently, bZIPs are well characterized in the model plants *Arabidopsis* and rice, and a total of 75 and 109 bZIPs have been identified, respectively [6, 10]. In *Arabidopsis*, the bZIP proteins AREB1, AREB2 and ABF3 cooperatively regulate the ABRE-dependent ABA signaling that is involved in drought resistance [11]. Overexpression of *Arabidopsis* bZIP gene *AtTGA4* confers enhanced drought resistance by improving nitrate transport and assimilation [12]. Contrarily, silencing of *AtbZIP24* using RNAi enhances salt tolerance in *Arabidopsis* [13]. In rice, qRT-PCR analysis showed that the transcription of most *OsbZIP* genes is upregulated by ABA and abiotic stress. Overexpression of *OsbZIP72* in rice displays a hypersensitivity to ABA, enhanced expression levels of ABA-responsive genes and improves drought resistance [14]. Overexpression of *OsbZIP23* and *OsbZIP71* enhances remarkably salt and drought tolerance through an ABA-dependent pathway in rice [15, 16]. Furthermore, overexpression of wheat *ABI-like* (*ABL*) bZIP TF gene *TaABL1* confers multiple stress tolerance against salt, drought and cold stresses [17]. Overexpression of *GhABF2* evidently enhances salt and drought tolerance both in *Arabidopsis* and cotton, while silencing *GhABF2* increases the sensibility to PEG and salt stresses [18].

Tomato has been one of the most widely produced crops worldwide because of its great nutritive and commercial values, and also one of the best-characterized model plants employed in genetic studies. However, for most tomato cultivars, salt produces adverse effects on tomato growth and development, such as decreased seed germination, inhibited growth and reduced fruit productivity [19]. Recently, the expression of 69 tomato *SlbZIPs* in various tissues, and the response to hormone and biotic stress have been reported [20]. However, the functions of most tomato bZIP genes in terms of the abiotic stress response remain unclear. The best-studied tomato bZIP gene, *SlAREB1*, is shown to play crucial functions in salt and drought tolerance. Meanwhile, it may act as a link of ABA signaling transduction to biotic stress responses [21–23]. Expression of several tomato bZIP genes such as *LebZIP2*, *FD*, *SlAREB2* and *ABZ1* is shown to be upregulated by salt, drought and wounding stresses or by organ-specific signals [22, 24–26]. Collectively, despite the involvement of bZIP TFs in various biological processes, the specific molecular functions of most tomato bZIP genes remain to be clarified.

Herein, we reported the functional characterization of *SlbZIP1* (Solyc01g079480.3, Genbank accession No. AF176641, formerly known as *LebZIP1*), it was then renamed as *SlbZIP04* and fell into Group A of bZIP TFs based on the systematic analysis of the bZIP TFs in tomato. In this study, we conducted a further functional analysis by silencing *SlbZIP1* gene in tomato to investigate its roles in salt and drought tolerance.

## Results

### Expression profiles of *SlbZIP1* in WT tomato and ripening mutant fruits

**Fig. 1:**
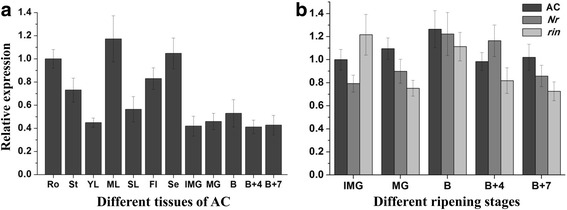
Expression profiles of *SlbZIP1* gene in different tissues of WT and ripening mutant fruits. **a** Expression patterns of *SlbZIP1* in different tissues of WT plants. The relative expression levels were normalized to 1 in roots. **b** Expression patterns of *SlbZIP1* between WT and ripening mutant fruits. Total RNA from the *Nr* (*never ripe*) and *rin* (*ripening inhibitor*) fruits from IMG to B + 7 equivalent stages with WT was subjected to qRT-PCR. The relative expression levels were normalized to 1 in IMG fruits of AC. Ro, roots; St, stems; Yl, young leaves; Ml, mature leaves; Sl, senescent leaves; Fl, flowers; Se, sepals; IMG, immature green; MG, mature green; B, breaker; B + 4, 4 d after breaker stage; B + 7, 7 d after breaker stage. Bars represent the mean of three biological replicates ± SE

The tissue-specific expression of *SlbZIP1* in WT tomato was quantified by qRT-PCR in various tissues. The results exhibited that *SlbZIP1* expression was relatively high in roots, mature leaves and sepals, but moderate to weak signals were observed in stems, flowers and fruits (Fig. 1a). To investigate whether there was any relationship between *SlbZIP1* and tomato fruit ripening mutants, *SlbZIP1* expression in *rin* and *Nr* mutants was also checked from IMG to B + 7 stage (equivalent stages with AC fruits). No distinct difference was detected among *rin*, *Nr* and WT fruits (Fig. 1b), suggesting that the expression of *SlbZIP1* is not affected by the single locus *RIN* and *Nr*.

### *SlbZIP1* expression was induced by multiple abiotic stress and hormone treatments

**Fig. 2:**
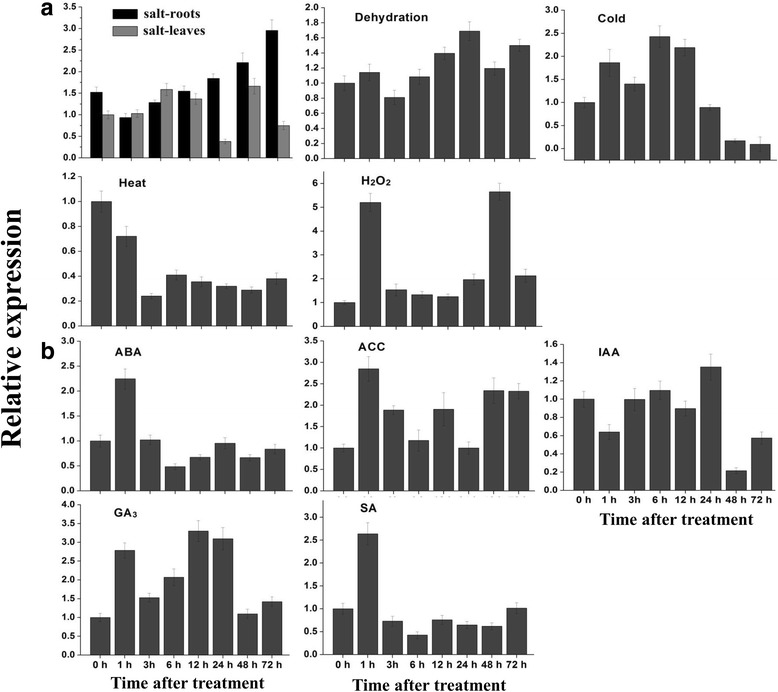
Expression profiles of *SlbZIP1* gene under various abiotic stress (**a**) and hormone (**b**) treatments. Gene expression was detected by qRT-PCR using total RNA from leaves or roots of WT plants. The relative expression levels were normalized to 1 in control plants (0 h). Bars represent the mean of three biological replicates ± SE

Although previous research has shown that *SlbZIP1* expression is upregulated by wounding and cold stresses, and ABA prevents its rapid wound-induced expression [27], the detailed response profiles of *SlbZIP1* gene to hormone and abiotic stress treatments have not been reported. Then a further expression analysis was carried out to speculate on its physiological and functional relevance by qRT-PCR. Consistent with the previous reports, upregulated *SlbZIP1* was observed under cold stress (4 °C), but downregulated expression was detected under heat stress (40 °C) (Fig. 2a). The transcript of *SlbZIP1* was also upregulated by NaCl (mainly in roots) and H_2_O_2_, while no significant change was detected when subjected to dehydration stress (Fig. 2a). Moreover, transcript levels of *SlbZIP1* increased with ABA, ACC, GA and SA treatments, while IAA treatment could not induce its transcript (Fig. 2b). The results indicate that *SlbZIP1* makes possible regulation on gene expression related to hormone and stress response in tomato.

### Silencing of *SlbZIP1* downregulates the expression of ABA biosynthesis- and signal transduction-related genes

**Fig. 3:**
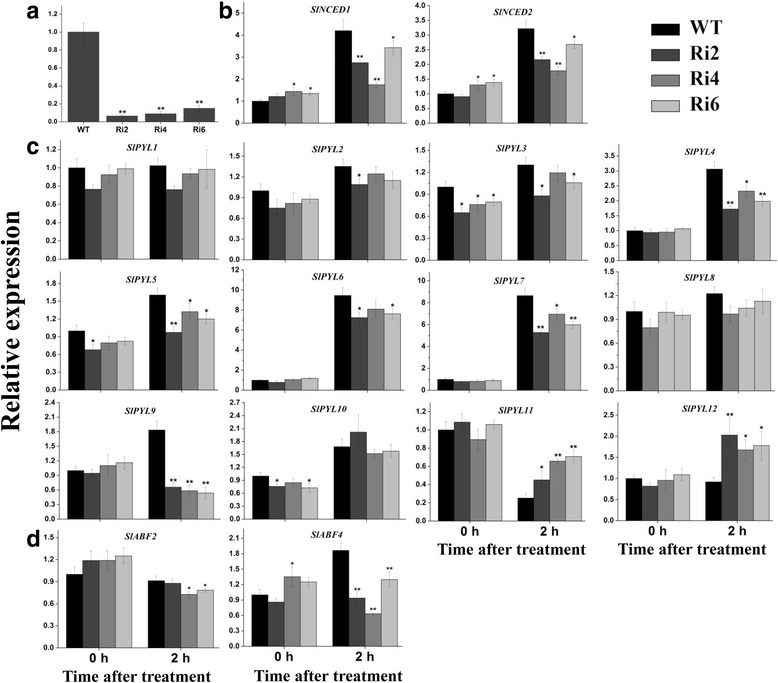
Expression profiles of a set of ABA biosynthesis- and signal transduction-related genes in *SlbZIP1*-RNAi plants. **a** Expression profiles of *SlbZIP1* in leaves of WT and *SlbZIP1*-RNAi lines. **b-d** Expression profiles of ABA biosynthesis genes *SlNCED1* and *SlNCED2* (**b**), ABA-dependent receptor genes *SlPYLs* (**c**), and ABA-responsive element binding factor (ABF) genes *SlABF2* and *SlABF4* (**d**) between WT and *SlbZIP1*-RNAi plants under normal and ABA-treated conditions. 8-week-old WT and transgenic plants were sprayed with 100 μM ABA solution, leaf samples were harvested before and 2 h post the ABA treatment. The relative expression levels were normalized to 1 in control plants (0 h). Bars represent the mean of three biological replicates ± SE. Asterisks indicate statistical significance (*0.01 < *P* < 0.05, **P < 0.01) between WT and transgenic plants, respectively

Since *SlbZIP1* was remarkably induced by ABA and salt stress, to examine its functional roles in greater depth, 9 independent *SlbZIP1*-RNAi lines were produced. The accumulation of *SlbZIP1* transcript was remarkably decreased to roughly 6–14% (Fig. 3a) of control levels in three RNAi lines (T3 generation, lines 2, 4 and 6), and thus they were chosen for further investigation. To verify specific repression of *SlbZIP1*, the expression levels of *SlbZIP07* (NM_001247576), *SlbZIP10* (AK327140) and *SlbZIP39* (AK326780) were monitored because they are the most similar (52.6, 45.0 and 69.3% similarity at the nucleotide level, respectively) tomato genes to *SlbZIP1*. The results showed that *SlbZIP10* and *SlbZIP39* mRNA levels were a little lower in transgenic plants than that of WT, while *SlbZIP07* mRNA remained unchanged (Additional file 1: Figure S1). We further conducted a multiple sequence alignment among *SlbZIP1*, *SlbZIP07*, *SlbZIP10* and *SlbZIP39*, the results indicate that the 435 bp DNA fragment of *SlbZIP1* used in the hairpin is specific (Additional file 2: Figure S2). The results indicate that *SlbZIP1* mRNA is specifically targeted by the *SlbZIP1-*RNAi transgene RNA.

The increased transcription of *SlbZIP1* under ABA treatment impelled us to detect whether silencing of *SlbZIP1* affects ABA biosynthesis and/or signal transduction in transgenic lines. The expression of *SlNCED1* and *SlNCED2* genes involved in ABA biosynthesis [28] was increased in transgenic plants grown under control conditions (0 h), while both of their expression were significantly downregulated in transgenic plants after 2 h of ABA treatment (Fig. 3b). Moreover, recent research conducted by ABA signaling shows that the primary ABA receptor is a central signaling module consisting of three classes of proteins, such as PYR/PYL/RCAR [29]. Then the transcription levels of 12 tomato PYL genes were detected between WT and transgenic plants. Wherein, no obvious difference in *SlPYL1*, *SlPYL2* and *SlPYL8* expression was detected for *SlbZIP1*-RNAi and WT plants under both conditions. On the contrary, the accumulation of *SlPYL3* and *SlPYL5* expression was reduced in different extents in transgenic plants under both conditions. Moreover, downregulated expression of *SlPYL4*, *SlPYL6*, *SlPYL7* and *SlPYL9*, and upregulated expression of *SlPYL11* and *SlPYL12* in transgenic plants were detected at 2 h after ABA treatment, while reduced *SlPYL10* expression was observed mainly under control conditions (Fig. 3c). Moreover, ABF (ABA-responsive element binding factors) genes are also the important downstream components of ABA signaling [30]. The transcription of two ABF genes, *SlABF2* and *SlABF4* was also markedly reduced in transgenic plants (Fig. 3d).

### Silencing of *SlbZIP1* significantly decreases salt tolerance

**Fig. 4:**
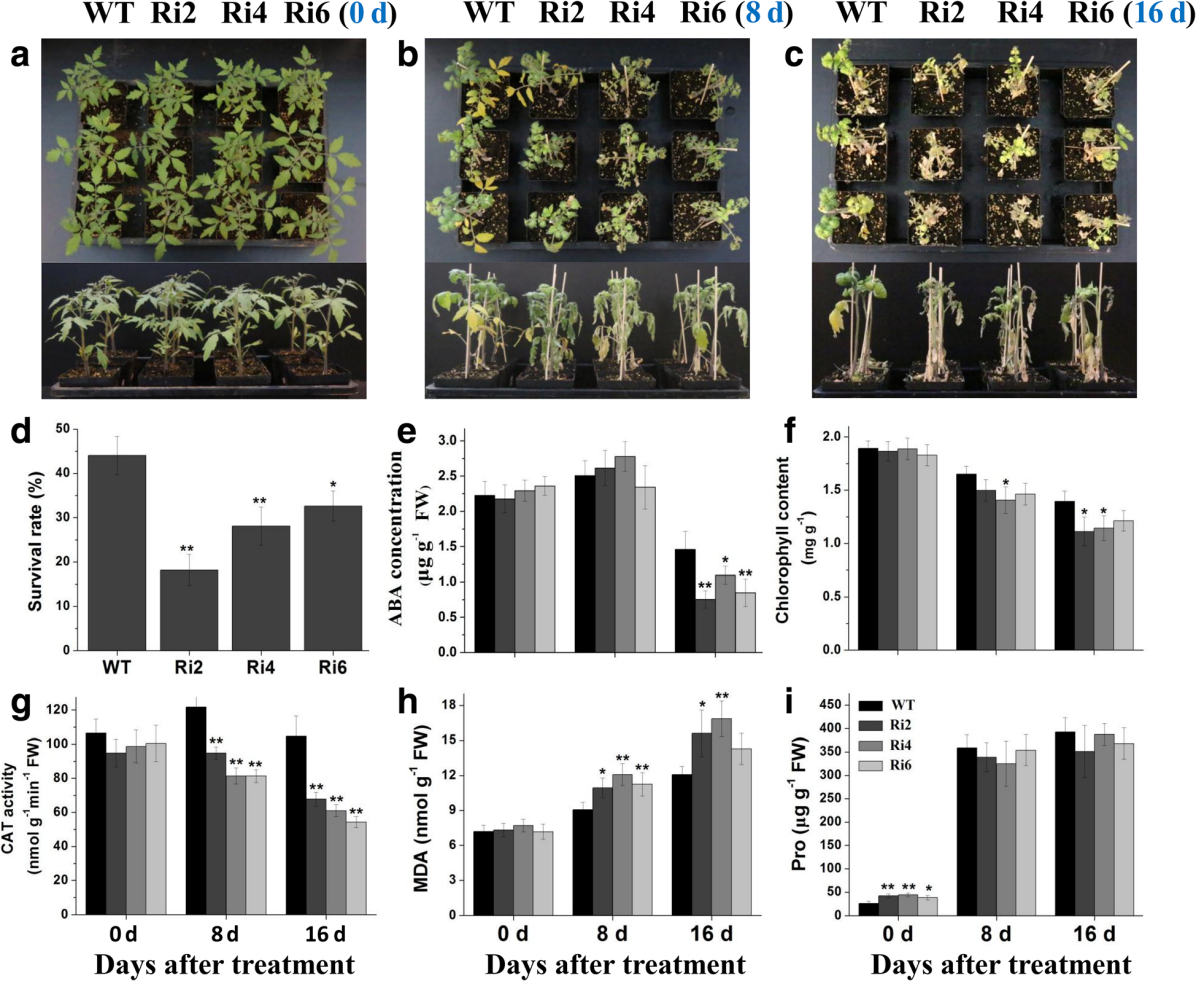
Salt stress tolerance of *SlbZIP1*-RNAi plants. Representative phenotypes of WT and transgenic tomato plants at 0 d (**a**), 8 d (**b**) and 16 d (**c**) after salt stress. 8-week-old plants were irrigated with 200 mL 400 mM NaCl every 72 h from the bottom of the pots. Representative plants are shown. **d** Survival rate of the plants shown after 16 d of salt stress and 5 d of recovery. Bars represent the mean of three biological replicates of 20 plants each. **e**-**i** Comparisons of ABA content (**e**), chlorophyll content (**f**), MDA content (**g**), CAT activity (**h**), and Pro content (**i**) between WT and transgenic plants at 0, 8 and 16 d after salt stress. Bars represent the mean of three biological replicates ± SE. Asterisks indicate statistical significance (*0.01 < *P* < 0.05, **P < 0.01) between WT and transgenic plants, respectively

Since tomato *SlbZIP1* gene responded to various abiotic stresses, the effects of salt stress on WT and *SlbZIP1*-RNAi tomato were investigated in soil. No abnormal morphological phenotype between WT and *SlbZIP1*-RNAi plants was observed under normal conditions (0 d, Fig. 4a, upper and lower). However, transgenic plants exhibited more notorious NaCl-induced symptoms under 400 mM NaCl, such as more wilting, especially in the lower leaves. The symptoms were more serious after 8 d of salt treatment with wilt of lower leaves in *SlbZIP1*-RNAi plants (Fig. 4b). Moreover, most of the leaves of transgenic plants displayed severe necrosis after 16 d exposed to salt stress, while the upper leaves of WT tomato showed less wilting at this time point (Fig. 4c). Moreover, after re-watering for 5 d, a lower survival rate of *SlbZIP1-*RNAi plants was observed (Fig. 4d).

To investigate the potential physiological mechanisms responsible for the reduced salt stress tolerance in *SlbZIP1-*RNAi plants, multiple stress-related physiological parameters were detected under normal and salt conditions. Changes were found in ABA, chlorophyll and malondialdehyde (MDA) contents, and CAT activity, which are typical parameters to evaluate plant stress tolerance. No obvious difference in these parameters was detected between WT and *SlbZIP1-*RNAi plants under normal conditions (0 d) except for proline levels (Figs. 4e-i). Upon exposure to salt stress, no distinct changes in ABA contents were observed between WT and transgenic plants at 8 d post-treatment, while ABA contents in *SlbZIP1-*RNAi plants were significantly lower than that of WT after 16 d of salt treatment (Fig. 4e). In addition, detectable reductions were also found in chlorophyll content (Fig. 4f) and CAT activity (Fig. 4g) in transgenic tomato leaves compared with WT plants. The MDA content was enhanced by salt stress, whereas the enhanced level in WT plants was lower than in transgenic lines (Fig. 4h). Furthermore, no significant change in proline contents was detected between WT and *SlbZIP1-*RNAi lines under salt stress (Fig. 4i). The data suggest that the expression of *SlbZIP1* correlates with the degree of salt tolerance in transgenic tomato.

### Silencing of *SlbZIP1* significantly reduces drought resistance

**Fig. 5:**
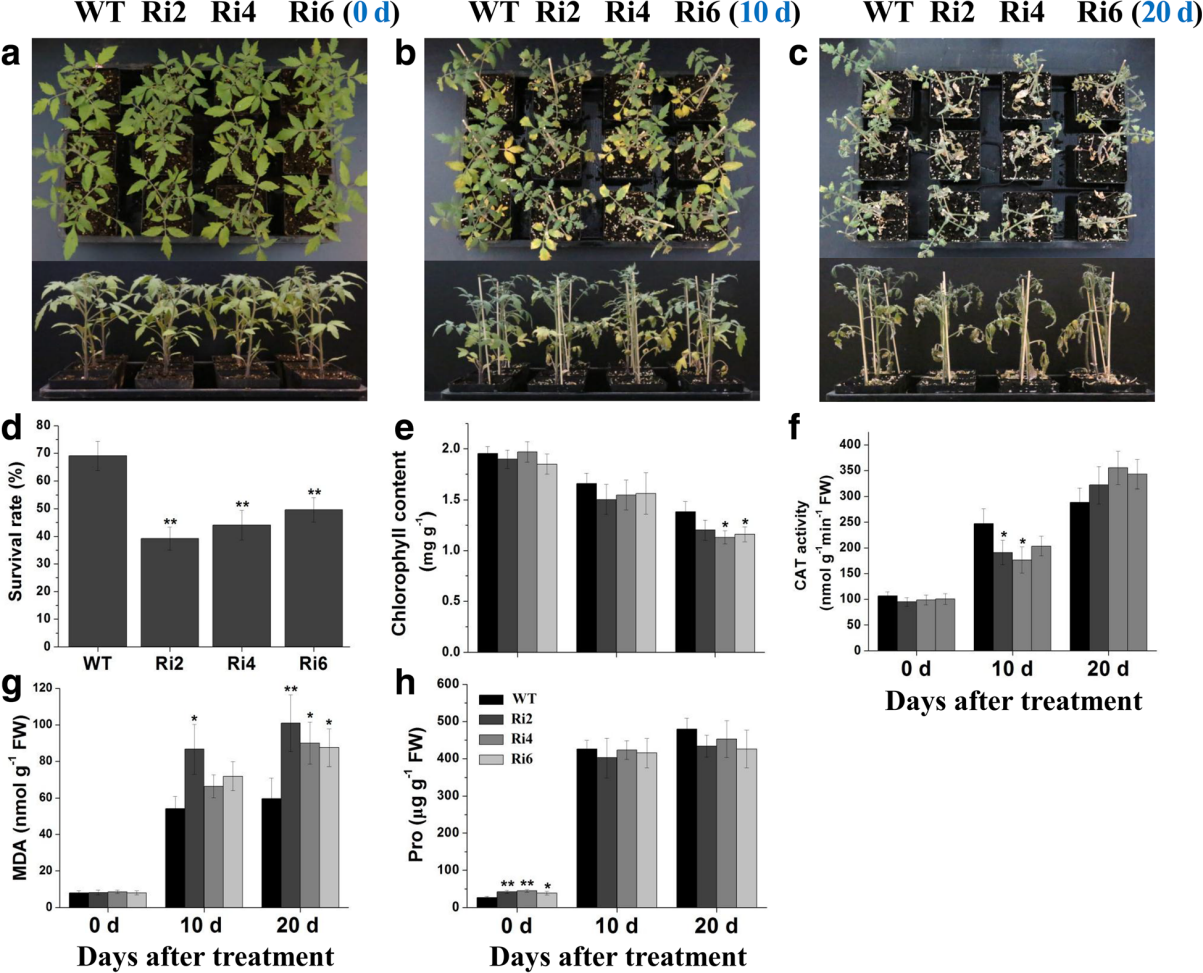
Drought tolerance testing of *SlbZIP1*-RNAi plants. 8-week-old WT and transgenic plants were incubated with 20% PEG6000 for up to 20 d. Representative phenotypes of WT and *SlbZIP1*-RNAi plants at 0 (**a**), 10 (**b**) and 20 d (**c**) after initiation of drought assay. **d** Survival rates of WT and transgenic plants after rewatering for 5 d following 20 d of PEG assay. Bars represent the mean of three biological replicates of 20 plants each. **e**-**h** Comparisons of chlorophyll content (**e**), CAT activity (**f**), MDA content (**g**), and Pro content (**h**) between WT and transgenic plants at 0, 10 and 20 d after drought stress. Bars represent the mean of three biological replicates ± SE. Asterisks indicate statistical significance (*0.01 < *P* < 0.05, **P < 0.01) between WT and transgenic plants, respectively

To further test the influences of *SlbZIP1* gene on drought resistance, a drought experiment was conducted by irrigated 20% PEG6000. Before the PEG stress (0 d), WT and *SlbZIP1-*RNAi plants displayed similar growth status (Fig. 5a, upper and lower). The leaves of both WT and transgenic plants began to exhibit leaf wilting with chlorophyll loss after 10 d of dehydration stress, while transgenic plants displayed more severe symptoms compared with control plants (Fig. 5b). Apparent difference was detected on the 20th day after dehydration treatment, most leaves of transgenic plants showed serious wilting, whereas WT plants were less affected (Fig. 5c). Moreover, a lower survival rate of *SlbZIP1-*RNAi plants than that of WT plants was observed after watering for 5 d (Fig. 5d). We also measured chlorophyll contents between WT and transgenic lines during the drought assay and found that, the chlorophyll reduction in transgenic lines was higher than that in WT plants both at 10 and 20 d post-treatment (Fig. 5e). And lower CAT activity in transgenic leaves compared with WT leaves was detected following the drought assay (Fig. 5f). Furthermore, the increase of MDA content in WT plants was lower than in transgenic lines during the post-drought treatment (Fig. 5g). In addition, no significant change in proline levels was detected between WT and *SlbZIP1-*RNAi plants under the same drought assay (Fig. 5h). The data indicate that *SlbZIP1* functions importantly in tomato drought resistance.

### Differentially expressed genes (DEGs) based on RNA-Seq data between WT and *SlbZIP1-*RNAi transgenic plants

**Fig. 6:**
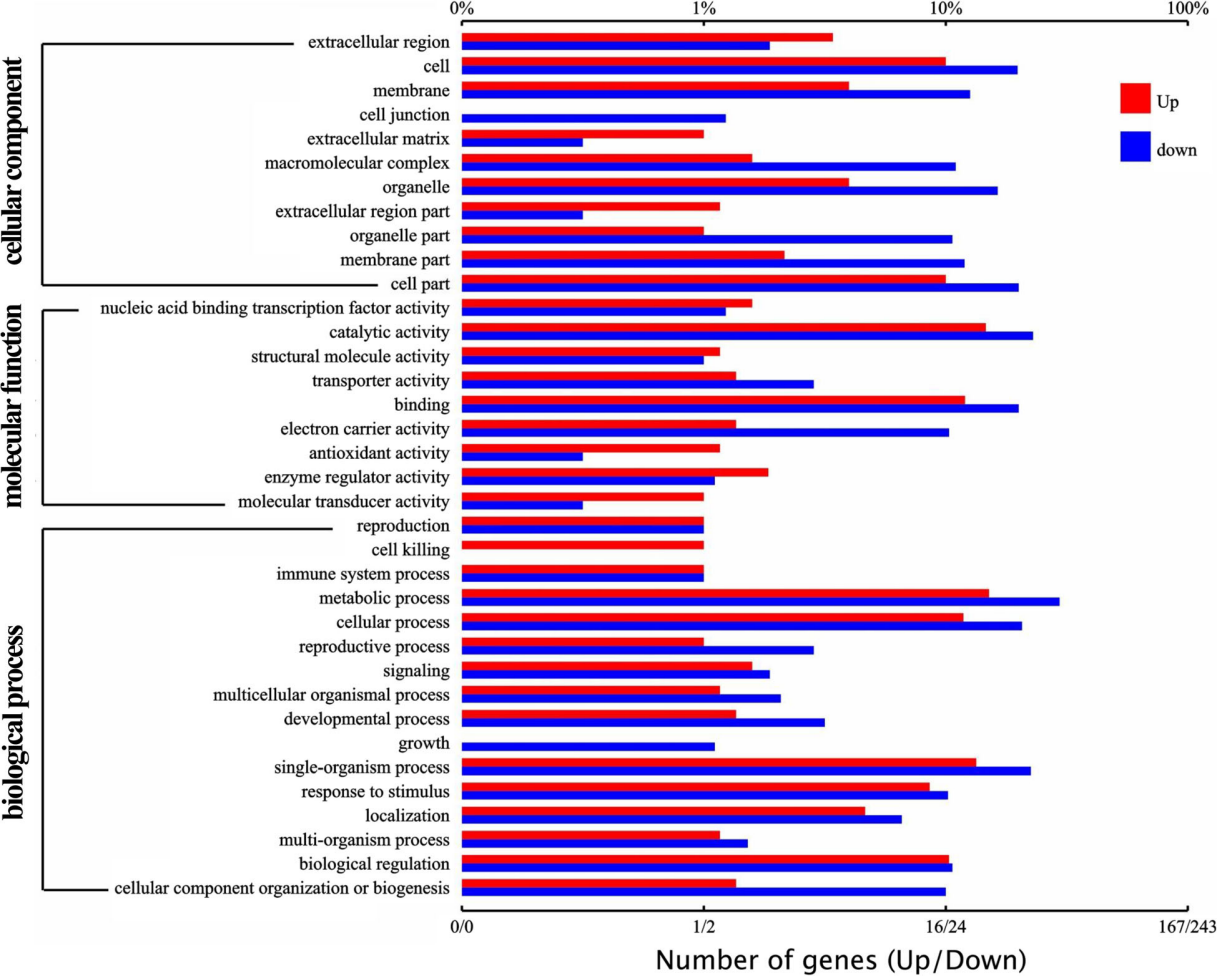
Functional analysis of DEGs in WT and *SlbZIP1*-RNAi plants based on RNA-Seq data. GO functional enrichment analysis of DEGs between WT and *SlbZIP1*-RNAi plants based on sequence homology. Top X axis and Bottom X axis represents to the numbers of upregulated or downregulated genes annotated by GO term and the percentages of annotated genes accounted for the total annotated genes, respectively. The Y axis refers GO classification including Cellular Component, Molecular Function, and Biological Process. The red bar and blue bar refers up-regulated genes and down regulated genes, respectively

To gain further insights into the potential mechanisms of the reduced salt and drought tolerance in *SlbZIP1-*RNAi lines, the gene expression between WT and transgenic lines (Ri2) treated with salt stress for 1 d was determined and compared using RNA sequencing. A total of 395 genes were identified as DEGs (280 downregulated and 115 upregulated) that exhibited a > twofold difference in expression in transgenic lines (Fig. 6, Additional file 3: Table S1), compared with the expression in WT plants. From the DEGs with function information, we found that over 50 genes are associated with biotic and abiotic stresses. The representative genes with reduced expression in leaf tissues are shown in Table 1. It is noteworthy that most of these genes encode proteins that respond to abiotic stress [e.g. MAP kinase kinase kinase (Solyc01g111880)], oxidative stress [e.g. peroxidase (Solyc08g069040)], biotic stimuli [e.g. pathogenesis-related proteins (e.g. Solyc02g031950 and Solyc04g007780), and protease inhibitor (e.g. Solyc03g020030 and Solyc03g020040)], and multiple TF genes [e.g. Trihelix TF (Solyc09g009250), Zinc finger protein TF (Solyc01g087050, Solyc05g025570 and Solyc01g067360) and MYB TF (Solyc11g065840, Solyc11g011050 and Solyc01g010910)] were also detected.

**Table 1:** RNA sequencing analysis of selected genes downregulated (*P* ≤ 0.05) in *SlbZIP1-*RNAi transgenic line Ri2 compared with WT tomato plants

Accession	Gene product	GO term	Log2 fold (Ri2/WT)
Solyc07g007450	Iron-sulfur cluster assembly protein	iron ion binding	−7.821905209
Solyc10g080580	Receptor-like kinase	protein phosphorylation	−7.371740136
Solyc02g067770	Cysteine-rich receptor-like protein kinase 2	protein phosphorylation	−7.212299904
Solyc02g082990	Stress-induced protein KIN2	Stress response	−7.12544498
Solyc11g064780	F-box/LRR-repeat protein	Disease resistance	−7.033024239
Solyc07g005040	H^+^-ATPase	ATP binding	−6.828267133
Solyc09g009250	Trihelix transcription factor PTL	Regulation of transcription	−6.828267133
Solyc02g086960	Receptor-like kinase	protein phosphorylation	−4.80337823
Solyc01g087050	Zinc finger protein WIP2-like	Regulation of transcription	−4.746313437
Solyc02g093540	Cytochrome P450	Controlling growth. Biotic stress	−4.369713338
Solyc01g010080	Potassium channel KAT3-like	potassium channel activity	−4.296148364
Solyc12g009190	Receptor-like protein kinase 3	protein phosphorylation	−4.136708132
Solyc11g065840	MYB transcription factor	Regulation of transcription	−3.719575489
Solyc05g047680	Cytochrome P450	Controlling growth. Biotic stress	−3.591453231
Solyc10g074440	Endochitinase	Stress response	−3.131124702
Solyc11g011050	MYB transcription factor	Regulation of transcription	−2.932906604
Solyc02g085130	Tubby-like F-box protein	Disease resistance	−2.871978777
Solyc09g084460	Wound-induced proteinase inhibitor 1	Stress response	−2.844760217
Solyc02g031950	Pathogenesis-related protein	Response to biotic stimulus	−2.815681677
Solyc05g025570	Zinc finger protein	Regulation of transcription	−2.69118912
Solyc08g069040	Peroxidase 1	Response to oxidative stress	−2.480134415
Solyc01g097630	F-box protein	Disease resistance	−2.411524305
Solyc08g013760	Putative F-box protein	Disease resistance	−2.33246778
Solyc01g010910	MYB transcription factor	Regulation of transcription	−2.319000953
Solyc00g011160	Cytochrome P450 like	Controlling growth. Biotic stress	−2.256203387
Solyc01g111880	MAP kinase kinase kinase 11	Biotic and abiotic stress response	−2.14925363
Solyc03g020030	Proteinase inhibitor II	Stress response	−2.146031647
Solyc01g066910	Lipid-transfer protein DIR1	Response to stress	−1.958613976
Solyc04g007780	Pathogenesis-related protein	Response to biotic stimulus	−1.762833313
Solyc10g050210	bZIP transcription factor	Regulation of transcription	−1.762190276
Solyc03g020040	Proteinase inhibitor II	Stress response	−1.707542181
Solyc01g067360	Zinc finger protein	Regulation of transcription	−1.701869583
Solyc03g034390	Lipid-transfer protein	Response to stress	−1.684560398

**Fig. 7:**
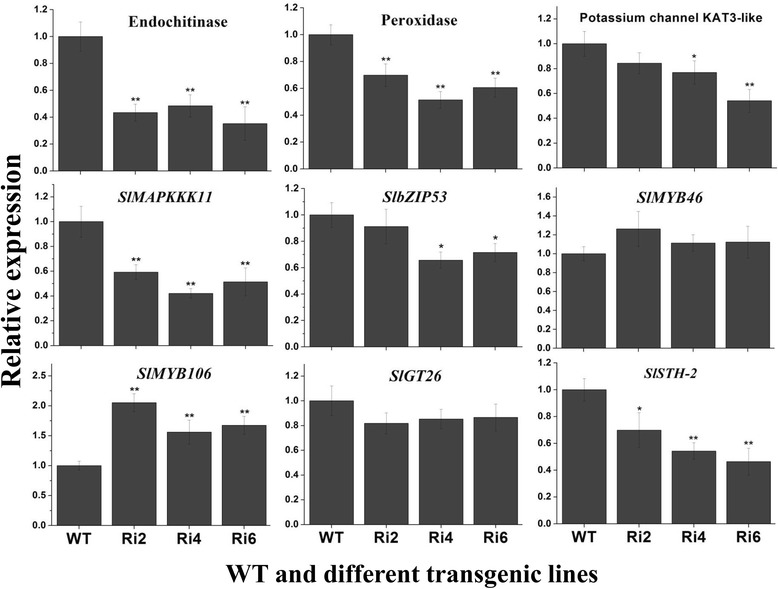
Transcript levels of selected biotic and abiotic stress-related genes in leaves of WT and *SlbZIP1*-RNAi plants under salt stress conditions. 8-week-old plants were irrigated with water containing 400 mM NaCl from the bottoms of the pots, then leaf samples were collected after 24 h. The relative expression levels were normalized to 1 in WT plants. Bars represent the mean of three biological replicates ± SE. Asterisks indicate statistical significance (*0.01 < *P* < 0.05, **P < 0.01) between WT and transgenic plants, respectively

Furthermore, 9 stress-related genes were selected and their expression was compared between WT and *SlbZIP1-*RNAi plants by qRT-PCR. With the exception of the elevated expression of *SlMYB106* (Solyc01g010910) [31] in transgenic plants, the expression of most genes selected for confirmation displayed expected transcript accumulations in transgenic plants, despite the changes of several genes exhibiting some differences among the transgenic plants (Fig. 7), supporting the outcomes of the RNA-seq analysis and the thesis that tomato SlbZIP1 is involved in regulating stress-related genes. Wherein, the transcription levels of stress-related endochitinase gene (Solyc10g074440) [32], peroxidase gene (Solyc08g069040) [33], potassium channel KAT3-like gene (Solyc01g010080) [34] and MAP kinase kinase kinase gene *SlMAPKKK11* (Solyc01g111880) [35] were downregulated remarkably in transgenic lines. The expression of bZIP TF gene *SlbZIP53* (Solyc10g050210) [20] was also reduced in transgenic plants. Nevertheless, no obvious difference in the expression of MYB TF gene *SlMYB46* (Solyc11g065840) [31] and Trihelix TF gene *SlGT26* (Solyc09g009250) [36] was observed. Furthermore, the transcription of a pathogenesis-related protein gene *SlSTH-2* (Solyc02g031950) [37] in *SlbZIP1-*RNAi plants was also downregulated (Fig. 7). These data indicate that silencing of *SlbZIP1* exerts influence on the transcription levels of a series of stress-related genes and hence results in reduced salt and drought tolerance in *SlbZIP1-*RNAi tomato.

## Discussion

The specific mechanisms of the ABA signaling network and stress responses in tomato, compared with the model plants *Arabidopsis* and rice remain largely unknown. bZIP TFs undoubtedly represent one of the most significant participants in the regulation of stress tolerance, which act pivotal roles in diverse physiological processes, including stress responses and post-stress recovery [3]. Though the participation of several tomato bZIP genes such as *SlAREB1, SlAREB2* [21, 22] and *LebZIP2* [26] in abiotic stress response has been reported, the biological roles of most tomato bZIPs remain unclear. Previously, expression analysis suggests that *SlbZIP1* transcript is induced by wounding, and ABA may act to curtail its wounding-induced synthesis [20, 27], whereas its roles in abiotic stress response remain undefined. In this study, we further confirmed that *SlbZIP1* expression was significantly improved by NaCl, cold and ABA (Fig. 2), suggesting that *SlbZIP1* may respond to various environmental cues. Furthermore, we presented the evidence that expression of Group A bZIP gene *SlbZIP1* correlated with the degrees of salt and drought tolerance testified by *SlbZIP1-*RNAi transgenic plants (Figs. 4 and 5). Furthermore, we determined that *SlbZIP1* was involved in regulating a variety of biotic and abiotic stress-related genes (Fig. 7, Additional file 3: Table S1). Recent works showed that group A of bZIPs, including *Arabidopsis* ABF1–4 and ABI5, and rice TRAB1, OsbZIP23, OsABF1 and OsABI5, participates in ABA-mediated and stress-related gene transcription [18], indicating that they may be involved in regulating ABA-dependent stress response pathways. Therefore, the conservation of group A bZIPs regulatory systems for ABA-dependent stress responses has been proposed.

The surveys that *SlbZIP1*-RNAi plants displayed reduced salt and drought tolerance as presented by lower survival ratio, worse growth performance and significantly reduced transcription of many stress-associated genes under salt and drought stresses, were presented. The results demonstrated that tomato SlbZIP1 functions as a positive regulator of salt and drought stress tolerance. Similarly, previously tomato *SlAREB1* was reported to play an essential role in improving salt and drought tolerance demonstrated by overexpression and silencing in transgenic tomato [22]. Besides, SlbZIP1 protein shares a close homolog with *Arabidopsis* bZIP53, the heterodimers of *Arabidopsis* bZIP53 and bZIP1 reprogram amino acid metabolism under low energy stress [38]. The possible mechanisms accounting for the changed stress tolerance in *SlbZIP1*-RNAi lines could be illustrated in part by multiple physiological parameters detected in this study. In general, as a crucial stress hormone, high degrees of ABA may accelerate and/or strengthen stress responses, which are associated with enhanced stress tolerance [1, 39]. Transgenic lines tended to accumulate less ABA than that of WT plants under salt stress (Fig. 4), which partly explained the reduced salt tolerance of transgenic plants. Proline acts as the regulator of antioxidant system or molecular chaperone to stabilize proteins [40], while it was not obviously changed in transgenic plants under both stress conditions (Figs. 4 and 5). Moreover, chlorophyll content is regarded as an indicator of the stress damage done to the photosynthetic apparatus. And stress conditions including salt and drought often generate ROS (reactive oxygen species) in plants, hence inducing damages to membrane lipids, proteins and nucleic acids. Therefore, enzymatic defending systems are adapted by plants for detoxifying ROS synthesized. Wherein, CAT is involved in the main defense system against H_2_O_2_ accumulation and toxicity [41–43]. This work exhibited that both chlorophyll content and CAT activity in *SlbZIP1*-RNAi lines were lower than that in WT under both stresses (Figs. 4 and 5). The reduced activity of antioxidant enzyme might underlie the higher H_2_O_2_ contents in *SlbZIP1*-RNAi plants. Furthermore, cell membranes are one of the primary targets of various environmental cues, and MDA is usually employed as an index of oxidative damages in plant [44, 45]. The present survey showed that MDA contents in transgenic plants were higher than that in WT tomato under both stresses (Figs. 4 and 5). In this context, increased MDA levels have also been detected in *SlAREB1*-antisense tomato plants [22]. These data suggest that *SlbZIP1*-RNAi plants have less robust photosynthetic abilities and more oxidative damage than WT plants, thus exhibiting reduced tolerance to salt and drought stresses.

Numerous molecular and biochemical studies have shown that ABA accumulates in various plant tissues to adapt to drought and salinity stresses, and is believed to act as an essential regulator in abiotic stress response [39, 46]. The results indicated that SlbZIP1 might act as a positive regulator in stress response by an ABA-mediated pathway. Firstly, treatment of exogenous ABA significantly affected the expression of *SlbZIP1* (Fig. 2), similar to previously reported ABA-responsive tomato *LebZIP2* gene, and its overexpression improves *NOA1* and *NR* transcription in the *N. benthamiana* leaves [26]. In this context, downregulated transcriptions of ABA biosynthesis-related genes including *SlNCED1* and *SlNCED2*, which are regarded as the rate-limiting step in the stress-induced ABA synthesis pathway [30, 47] were detected in *SlbZIP1*-RNAi plants (Fig. 3). The result is similar to the data that the overexpression of grape *VlbZIP36* gene obviously improves *AtNCED3* expression in transgenic *Arabidopsis* [48]. The endogenous ABA content of *SlbZIP1*-RNAi lines was also remarkably lower than that of WT exposed to salt treatment (Fig. 4e). Furthermore, the expression of most of detected tomato PYL genes encoding ABA-dependent receptors and two ABF genes *SlABF2* and *SlABF4* were also downregulated in *SlbZIP1*-RNAi plants (Fig. 3). The data indicate that silencing of *SlbZIP1* may affect ABA biosynthesis/signal transduction through regulating directly or indirectly the relevant gene expression.

The transcriptions of a series of biotic- and abiotic stress-related genes were differentially expressed in *SlbZIP1*-RNAi lines (Additional file 3: Table S1, Fig. 7). The transcription of stress-related genes leads to alterations of biochemical and physiological pathways that are pivotal for plants adaptation to unfavorable conditions. In this study, remarkably downregulated expression of multiple stress-relevant genes, including endochitinase gene [32], peroxidase gene [33], potassium channel KAT3-like gene [34] and *SlMAPKKK11* gene [35] were detected in transgenic lines. The stress-related TF gene *SlbZIP53* was also reduced in transgenic plants, and it has been well documented that bZIP TFs participate in diverse biotic and abiotic stress responses in multiple plant species [20]. Interestingly, the transcription levels of a pathogenesis-relevant gene *SlSTH-2* [37], were also reduced in *SlbZIP1-*RNAi plants. Thus, the results indicate that *SlbZIP1*, besides playing a role in tomato adaptation to abiotic stresses, may also integrate signals derived from both biotic and abiotic stresses, while further clarification is required. Thus, the decreased tolerance to salt and drought assays detected in the present study can be, at least partially, associated with SlbZIP1-regulated downstream genes. The results are in line with previous studies, in which improved tolerance to stress is attributed to upregulated expression of multiple biotic and abiotic stress-relevant genes in the *LebZIP2-* and *SlAREB1*-overexpressing lines [5, 22, 26]. In addition, since ABA contents in plants are improved in response to abiotic stress such as salt and drought, resulting in transcription of stress-related genes, thus the reduced ABA accumulation in *SlbZIP1-*RNAi plants will restrict ABA-dependent pathways that regulate the expression of many stress-related genes [39, 49]. Collectively, we suppose that *SlbZIP1* mediates signaling pathways to cope with abiotic stress through modulating the transcription of stress-responsive genes, thus silencing of *SlbZIP1* resulting in a reduced salt and drought tolerance in *SlbZIP1-*RNAi lines. Combining these results, we propose a hypothetical molecular mechanism for *SlbZIP1* functions in salt and drought stress tolerance in tomato, as the scheme representing in Additional file 4: Figure S3.

## Conclusions

Collectively, the data presented in this study not only reveal an important regulatory function for tomato SlbZIP1 in salt and drought stress tolerance, but also provide a foundation for further investigation of salt- and drought-induced signaling pathways in which SlbZIP1 participates. Thus SlbZIP1 may hold a potential application in the engineering of salt- and drought-tolerant cultivars. However, functional investigation of *SlbZIP1*-overexpressioning transgenic plants is necessary to further determine the functions of tomato *SlbZIP1* in stress tolerance. Moreover, further explorations on the identification of *SlbZIP1*-regulated downstream genes will be useful to clarify the mechanisms of *SlbZIP1* in the regulation of salt and drought stress tolerance.

## Methods

### Plant materials and growth conditions

The WT tomato (*Solanum lycopersicom* Mill. cv. Ailsa Craig) and transgenic seeds (T3 generation) were sterilized before germinating and the seedlings were then sown in commercially sterilized soil mix (peat moss, perlite, and vermiculite, 3:1:1, *v*/v/v). The plants were cultivated in a greenhouse under sodium lights timed for 16 h days (27 °C) and 8 h nights (18 °C). The tissues including roots, stems, flowers, and leaves and fruits of individual period were gathered as described before [50]. All the plant samples were collected and immediately frozen in liquid nitrogen and stored at − 80 °C.

### Plant abiotic stress and hormone treatments

Tomato seedlings were grown in a greenhouse under the same conditions mentioned above, and all the treatment experiments were conducted using potted 35 day-old plants chose according to their uniformity. Plants were irrigated with a 1 g L^− 1^ Murashige and Skoog nutrient solution once per week. Untreated plants were used as controls, and leaves and roots were collected at 1, 3, 6, 12, 24, 48, 72 h after each treatment. In each case, individual plants were used for each timepoint and treatment in triplicate and a total of 21 plants were employed in each treatment. Salt stress was conducted by submerging the roots of plants in 200 mM NaCl, then leaves and roots were harvested. For dehydration essay, plants were gently pulled out and washed carefully with water to remove soil, and left on a piece of dry filter paper at 25 ± 1 °C, and then the leaf samples were collected. “Cold” and “heat” stress essays were applied by incubating the plants at 4 and 40 °C, respectively [51]. For oxidative stress, the plants were sprayed with 10 mM H_2_O_2_ solution. For hormone treatments, the plants were sprayed with 0.1 mM ABA, ACC, GA_3_, IAA, and 2 mM SA solution and left for 1, 3, 6, 12, 24, 48, 72 h at 25 ± 1 °C, and then the leaves were collected. All the collected samples were frozen immediately in liquid nitrogen and stored at − 80 °C.

### RNA extraction and qRT-PCR analysis

Total RNA was extracted by the Trizol reagent (Invitrogen, Shanghai, China). 2 μg RNA was reverse-transcribed using PrimeScript reverse transcriptase (with gDNA Eraser for Perfect Real Time) (TaKaRa, Dalian, China) with the mix of Oligo dT Primer and Random 6 mers. The qRT-PCR reaction consisted of 3.2 μL distilled water, 5 μL 2 × SYBR *Premix Ex Taq* II (Tli RNaseH Plus) (TaKaRa, Dalian, China), 0.4 μL 10 mM each primer and 1 μL cDNA. qRT-PCR was conducted using the CFX96™ Real-Time System (Bio-Rad, USA) under the procedures below: 95 °C 30 s, 40 cycles of 95 °C 5 s, 60 °C 1 min, followed by a melting curve analysis. Tomato *CAC* gene and *EF1α* gene were selected as the internal standard for organ expression and abiotic stress analysis, respectively [52, 53].

### Construction of RNAi vector and tomato transformation

The *SlbZIP1*-RNAi vector was constructed using the pBIN19 driven by the CaMV 35S promoter (Additional file 5: Figure S4). The 435 bp *SlbZIP1*-specific fragment was amplified using the primers *SlbZIP1*-RNAi-F/R (Additional file 6: Table S2), and confirmed by sequencing. The obtained construct was transformed into WT tomato (Ailsa Craig) by *Agrobacterium tumefaciens* (LBA4404)-mediated transformation of cotyledon explants, and transformed plants were selected for kanamycin resistance (50 mg L^− 1^). DNA was isolated based on the method of Genomic DNA Extraction Kit (Invitrogen, Shanghai, China), and then analyzed by PCR to ascertain the presence of T-DNA using the primers *NPTII*-F/R (Additional file 6: Table S2).

### Phenotype analyses for salt and drought stress tolerance and ABA treatment assays

WT and *SlbZIP1*-RNAi plants were pre-grown in seedling nurseries for 14 d and transferred to pots in the greenhouses under the conditions described above. Then 8-week-old plants of similar size were selected for salt and drought tolerance assays. The plants were irrigated from the bottom of the pots with 400 mM NaCl (200 mL) or 20% PEG6000 (200 mL) every 72 h. Survival rate was calculated after 5 d of recovery from both stresses. Plants were considered as survivals if there were healthy and green young leaves after 5 d of rewatering. For the ABA treatment of the transgenic plants, the plants of similar size were sprayed with 100 μM ABA solution and left for 2 h at 25 ± 1 °C, while untreated plants were used as controls.

### Evaluation of salt stress tolerance

ABA was quantified using a high-performance liquid chromatography (HPLC, Rigol L3000), ABA measurements were conducted as described before [54]. The leaf samples of soil-grown WT and *SlbZIP1-*RNAi plants under normal and salt treatment for 8 d and 16 d were used for ABA measurement. Thermo solution 220 spectrophotometer was used for physiological parameter measurements at 25 °C. The protocols for determination of chlorophyll (Chl) and malondialdehyde (MDA) content were as published previously [55]. Detection of CAT activities was assayed based on L Góth [56]. The reactions were initiated by adding H_2_O_2_ solution, and the activities were monitored by detecting the reduction in the absorbance of H_2_O_2_ at 240 nm. Free proline contents in tomato leaves were detected as previously described [57].

### RNA sequencing and data analysis

Eight-week-old WT and *SlbZIP1*-RNAi plants (mixed samples of six plants) were irrigated with water containing 400 mM NaCl from the bottoms of the pots, and then leaf samples were collected after 24 h. Then RNAs extracted from the salt-treated leaves were employed for the RNA sequencing using Illumina HiSeq 2500 platform, and the 150 bp paired-end reads were generated by Genepioneer Bioinformatics Institute (Nanjing, China). The clean reads were filtered from the raw reads by removing duplication sequences, adaptor sequences and low-quality reads. Cleaned reads were subsequently mapped to the tomato reference genome by HISAT2 software [58]. The quantitative information of the RNA-seq data can be seen in Additional file 7: Table S3. Gene expression abundance was analyzed using StringTie software [59], to identify DEGs (differentially expressed genes), transcript abundance was normalized by FPKM (expected number of Fragments Per Kilobase of transcript sequence per Millions base pairs sequenced) method, and FDR (false discovery rate) ≤ 0.05 was used to determine the threshold for DEGs. Then qRT-PCR was carried out to validate the results of RNA-seq, and gene-specific primers used for qRT-PCR of selected stress-related genes were listed in Additional file 6: Table S2.

### Statistical analysis

When appropriate, the results were analyzed by one-way analysis of variance (ANOVA) and means different were significant by a Dunnett’s test at *P* < 0.05 and *P* < 0.01. Statistical analyses were conducted with SPSS software 20 version (IBM Corp., USA). In addition, we used a cut-off value of threefold for tissue-specific transcription and twofold for stress induction or repression [60].

## Additional files

Additional file 1: **Figure S1.** Relative expression profiles of *SlbZIP07*, *SlbZIP10* and *SlbZIP39* in the leaves of WT and *SlbZIP1*-RNAi lines under normal conditions. (DOCX 77 kb)

Additional file 2: **Figure S2.** Multiple sequence alignment among *SlbZIP1*, *SlbZIP07*, *SlbZIP10* and *SlbZIP39* genes. (DOCX 570 kb)

Additional file 3: **Table S1.** Gene products of annotated genes or with sequence similarity showing at least 2-fold change in transcript abundance (*p* < 0.05) in leaves of *SlbZIP1*-RNAi line Ri2 compared with WT plants. (XLSX 76 kb)

Additional file 4: **Figure S3.** Proposed model depicting the functions of *SlbZIP1* in the regulation of salt and drought stress tolerance. (DOCX 102 kb)

Additional file 5: **Figure S4.** Hairpin construct of the *SlbZIP1* gene for double-stranded RNAi vector. (DOCX 96 kb)

Additional file 6: **Table S2.** Specific primer sequences used for cloning procedure and qRT-PCR analysis. (DOCX 18 kb)

Additional file 7: **Table S3.** The quantitative information of the RNA-seq data. (DOCX 17 kb)

## Abbreviations

ABA: Abscisic acid
ABF: ABRE binding factor
ABRE: ABA responsive element
ACC: 1-aminocyclopropane-1-carboxylate
bZIP: Basic leucine zipper
CAT: Catalase
GA: Gibberellin acid
IAA: Indole-3-acetic acid
MDA: Malondialdehyde
*Nr*: N*ever ripe*
Pro: Proline
qRT-PCR: quantitative reverse transcription-PCR
*rin*: R*ipening inhibitor*
ROS: Reactive oxygen species
SA: Salicylic acid
TF: Transcription factor
WT: Wild-type
