# Supplementary material for: Basic leucine zipper transcription factor SlbZIP1 mediates salt and drought stress tolerance in tomato
Source: BMC Plant Biol. 2018 May 8;18:83. doi: 10.1186/s12870-018-1299-0 (PMC5941487; doi:10.1186/s12870-018-1299-0)
Supplement: Supplementary file 1 — Figure S1. Relative expression profiles of SlbZIP07, SlbZIP10 and SlbZIP39 in the leaves of WT and SlbZIP1-RNAi lines under normal conditions. (DOCX 77 kb) [file 12870_2018_1299_MOESM1_ESM.docx]

**
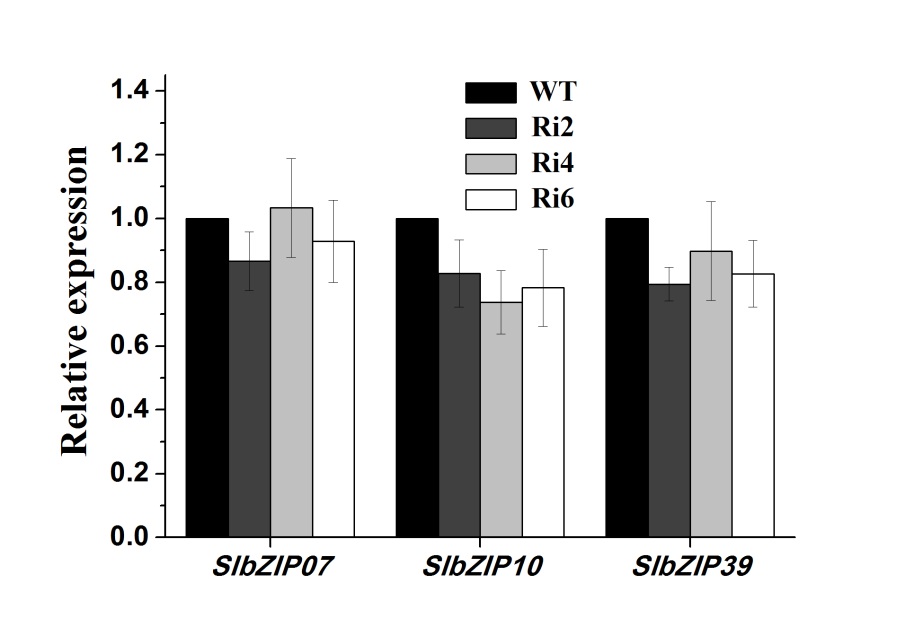
**

**Additional file 1: Figure S1**. Relative expression profiles of *SlbZIP07*, *SlbZIP10* and *SlbZIP39* in the leaves of WT and *SlbZIP1*-RNAi lines under normal conditions. The relative expression levels were normalized to 1 in WT plants. Bars represent the mean of three biological replicates ± SE.
