# Supplementary material for: Basic leucine zipper transcription factor SlbZIP1 mediates salt and drought stress tolerance in tomato
Source: BMC Plant Biol. 2018 May 8;18:83. doi: 10.1186/s12870-018-1299-0 (PMC5941487; doi:10.1186/s12870-018-1299-0)
Supplement: Supplementary file 2 — Figure S2. Multiple sequence alignment among SlbZIP1, SlbZIP07, SlbZIP10 and SlbZIP39 genes. (DOCX 570 kb) [file 12870_2018_1299_MOESM2_ESM.docx]

**
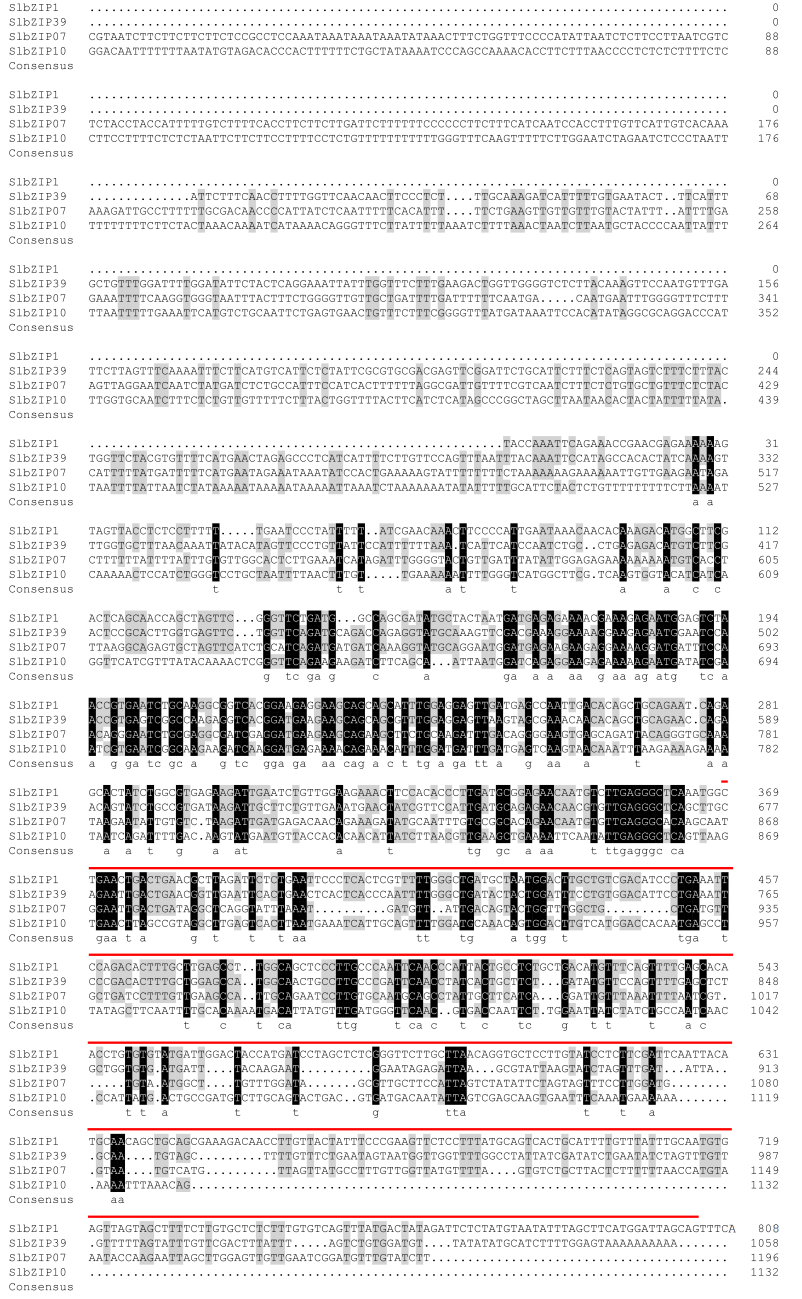
Additional file 2:** **Figure S2**. Multiple sequence alignment among *SlbZIP1*, *SlbZIP07*, *SlbZIP10* and *SlbZIP39* genes. The 435 bp DNA fragment of *SlbZIP1* used in the hairpin is indicated on top of the sequences.
