# Supplementary material for: Basic leucine zipper transcription factor SlbZIP1 mediates salt and drought stress tolerance in tomato
Source: BMC Plant Biol. 2018 May 8;18:83. doi: 10.1186/s12870-018-1299-0 (PMC5941487; doi:10.1186/s12870-018-1299-0)
Supplement: Supplementary file 4 — Figure S3. Proposed model depicting the functions of SlbZIP1 in the regulation of salt and drought stress tolerance. (DOCX 102 kb) [file 12870_2018_1299_MOESM4_ESM.docx]

**
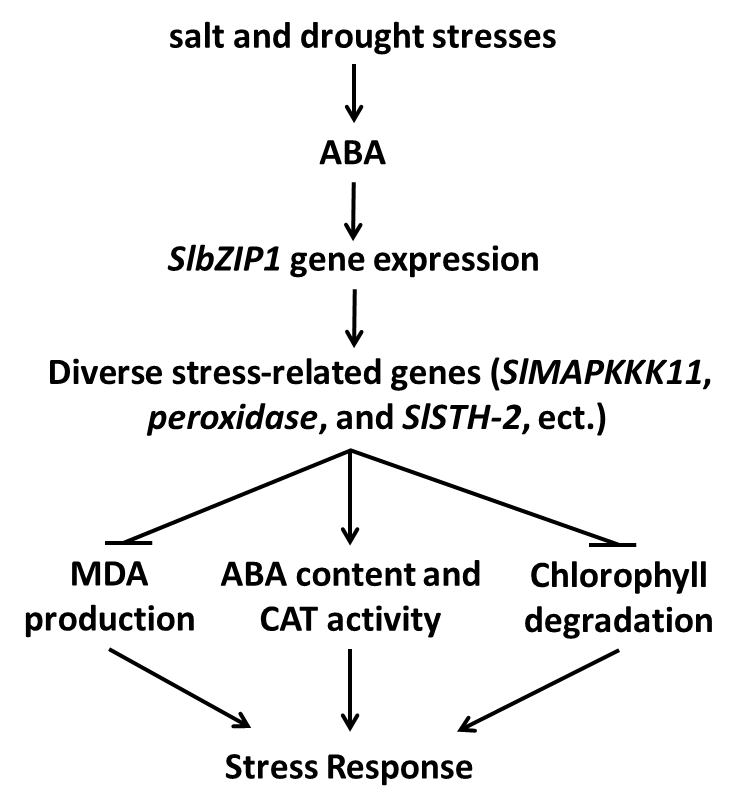
Additional file 4: Figure S3.** Proposed model depicting the functions of *SlbZIP1* in the regulation of salt and drought stress tolerance.
