# Supplementary material for: Basic leucine zipper transcription factor SlbZIP1 mediates salt and drought stress tolerance in tomato
Source: BMC Plant Biol. 2018 May 8;18:83. doi: 10.1186/s12870-018-1299-0 (PMC5941487; doi:10.1186/s12870-018-1299-0)
Supplement: Supplementary file 5 — Figure S4. Hairpin construct of the SlbZIP1 gene for double-stranded RNAi vector. (DOCX 96 kb) [file 12870_2018_1299_MOESM5_ESM.docx]

**
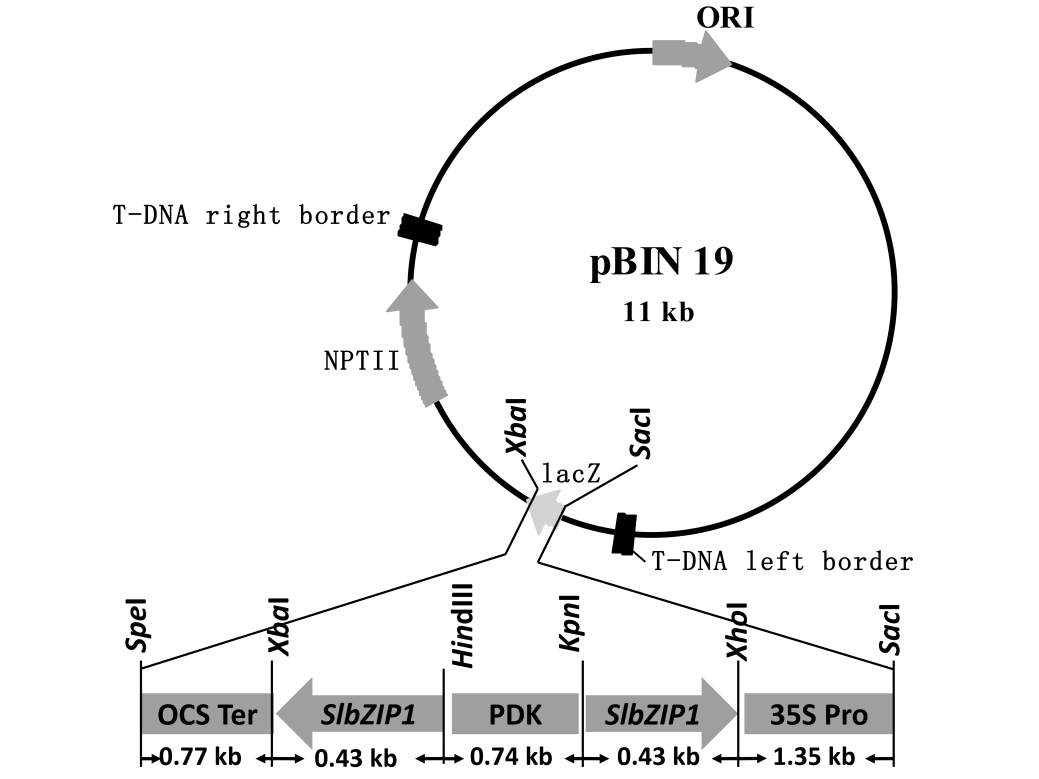
**

**Additional file 5: Figure S4.** Hairpin construct of the *SlbZIP1* gene for double-stranded RNAi vector. The *SlbZIP1* gene-specific sequence in the antisense and sense orientations were linked with a *PDK* gene fragment and as a transcriptional unit for hairpin RNA expression which controlled by the CaMV 35S promoter and terminated by the 35S terminator. Among which, *Spe*I and *Xba*I are isocaudamers.
