# Supplementary material for: Basic leucine zipper transcription factor SlbZIP1 mediates salt and drought stress tolerance in tomato
Source: BMC Plant Biol. 2018 May 8;18:83. doi: 10.1186/s12870-018-1299-0 (PMC5941487; doi:10.1186/s12870-018-1299-0)
Supplement: Supplementary file 6 — Table S2. Specific primer sequences used for cloning procedure and qRT-PCR analysis. (DOCX 18 kb) [file 12870_2018_1299_MOESM6_ESM.docx]

**Additional file 6: Table S2**. Specific primer sequences used for cloning procedure and qRT-PCR analysis.

| Primer sequences (5' →3') | | Application | | |
| --- | --- | --- | --- | --- |
| *SlbZIP1*-RNAi-F | CGGGGTACCAAGCTTCTGAACTGACTGAACGCTTAGAT | | To establish *SlbZIP1* RNAi lines; added *Kpn* I and *Hin*d III site underlined |  |
| *SlbZIP1*-RNAi-R | CCGCTCGAGTCTAGACTGCTAATCCATGAAGCTAAATAT | | To establish *SlbZIP1* RNAi lines; added *Xho* I and *Xba* I site underlined |  |
| *NPTII*-F | GACAATCGGCTGCTCTGA | | Positive transgenic plants detection |  |
| *NPTII*-R | AACTCCAGCATGAGATCC | |  |  |
| *CAC*-Q-F | CCTCCGTTGTGATGTAACTGG | | Internal standard gene for qRT-PCR in tomato development |  |
| *CAC*-Q-R | ATTGGTGGAAAGTAACATCATCG | |  |  |
| *EF1α*-Q-F | TACTGGTGGTTTTGAAGCTG | | Internal standard gene for qRT-PCR under abiotic stress |  |
| *EF1α*-Q-R | AACTTCCTTCACGATTTCATCATA | |  |  |
| *SlbZIP1-Q-F* | GGGCTGATGCTAATGGACTTG | | qRT-PCR analysis for *SlbZIP1* |  |
| SlbZIP1-Q-R | GCAGAGGCAGTAATGGGTTGA | |  |  |
| SlbZIP07-Q-F | AATGGAATTGACTGATAGGCTCAG | | qRT-PCR analysis for SlbZIP07 |  |
| SlbZIP07-Q-R | GAAGCAACGCTATCCAAACAAG | |  |  |
| SlbZIP10-Q-F | GTGAACTTAGCCGTAGGCTTGAG | | qRT-PCR analysis for SlbZIP10 |  |
| SlbZIP10-Q-R | CATAATGGGTTGATTGGCAGATAG | |  |  |
| SlbZIP39-Q-F | TTTGGGCTGATACTACTGGATTTC | | qRT-PCR analysis for SlbZIP39 |  |
| SlbZIP39-Q-R | TCATCACACCAGCAGAGCTCA | | qRT-PCR analysis for stress-related genes |  |
|  |  | |  |  |
| SlASR5-Q-F | ACAACATCACCGTTTGTTCCAC | |  |  |
| SlASR5-Q-R | TGGAGATGGCTATGGTGCTTTA | |  |  |
| MYB-Q-F | TGGACACGAACGAGCAACAC | |  |  |
| MYB-Q-R | AATTTCTCCCTGCCCTTTAGC | |  |  |
| SlHsp23.8-Q-F | CATCACTATTCAACAGGCTCGTC | |  |  |
| SlHsp23.8-Q-R | GGTCAACATCAACGCCACG | |  |  |
| SlPR4-Q-F | GCGGTAGATGCTTGAGGGTG | |  |  |
| SlPR4-Q-R | CTGATAGCCCAATCCATTAGTGTC | |  |  |
| SlNAC43-Q-F | ATGGACAACACCTCCCGAATC | |  |  |
| SlNAC43-Q-R | GCTGCTTCCAATCTGGTGTGA | |  |  |
| SlNAC56-Q-F | TCAAGATAGGGAAGCAAGAGGA | |  |  |
| SlNAC56-Q-R | ACCATTGTTCTTTTGCATCCA | |  |  |
| SlWRKY6-Q-F | TGGGACCATAGTTCCAAGACAA | |  |  |
| SlWRKY6-Q-R | GTTGTTCCGAGGTGATCCAGA | |  |  |
| SlWRKY28-Q-F | CATCATTGTCCAGCAACTCTTAGG | |  |  |
| SlWRKY28-Q-R | TTGGGAAAATAGTTGTGGAGGAA | |  |  |
| SlWRKY75-Q-F | CTAATGGCAAGCATGGAAACTC | |  |  |
| SlWRKY75-Q-R | ATAGCTCCTTGGGAATTTGTTGT | |  |  |
