# Supplementary material for: Basic leucine zipper transcription factor SlbZIP1 mediates salt and drought stress tolerance in tomato
Source: BMC Plant Biol. 2018 May 8;18:83. doi: 10.1186/s12870-018-1299-0 (PMC5941487; doi:10.1186/s12870-018-1299-0)
Supplement: Supplementary file 7 — Table S3. The quantitative information of the RNA-seq data. (DOCX 17 kb) [file 12870_2018_1299_MOESM7_ESM.docx]

**Additional file 7: Table S3**. The quantitative information of the RNA-seq data.

| Samples | Trimmed reads | Mapped reads | Alignment % | Transcript number | Gene counts |
| --- | --- | --- | --- | --- | --- |
| WT | 37370308 | 23687679 | 78.88% | 30551 | 19229 |
| Bi2 | 37073065 | 20607737 | 89.95% | 30634 | 19145 |
